# Resection of infarcted upper lobe with reimplantation of the lower lobe allograft after initial bilateral full-sized lung transplantation: a case report

**DOI:** 10.1016/j.jhlto.2025.100295

**Published:** 2025-05-22

**Authors:** An-Lies Provoost, Paul De Leyn, Hans G.L. Van Veer, Lieven P. Depypere, Yanina Jansen, Robin Vos, Birgit Weynand, Sophie Pirenne, Dirk E. Van Raemdonck, Laurens J. Ceulemans

**Affiliations:** a Department of Thoracic Surgery, University Hospitals Leuven, Leuven, Belgium; b Laboratory of Respiratory Diseases and Thoracic Surgery (BREATHE), Department of Chronic Diseases and Metabolism, KU Leuven, Leuven, Belgium; c Department of Respiratory Medicine, University Hospitals Leuven, Leuven, Belgium; d Department of Pathology, University Hospitals Leuven, Leuven, Belgium

**Keywords:** Anastomotic complications, Lobar-lung auto-reimplantation, Lung transplantation

## Abstract

We describe the case of a patient with lobar-lung auto-reimplantation following bilateral lung transplantation because of vascular and bronchial anastomotic complications with upper lobe infarction. Pneumonectomy of the left allograft with ex-vivo upper lobectomy and lower lobe reimplantation was successfully performed, resulting in favorable short-term recovery and improving graft function at 7-month follow-up.

## Background

Anastomotic complications following lung transplantation (LTx) can have detrimental consequences like infarction, abcedation, embolization with stroke, and in severe cases, death. While isolated atrial cuff, pulmonary artery (PA), or bronchial stenosis may be treated with minimally invasive approaches,^1^, ^2^, ^3^ complications at multiple anastomoses require more extensive surgical intervention. We present a case of lobar-lung auto-reimplantation due to combined bronchial and vascular anastomotic problems with left upper lobe (UL) infarction.

## Case report

A 42-year-old female underwent bilateral LTx without extra corporeal life support (ECLS) by anterolateral thoracotomy for Langerhans histiocytosis and pulmonary hypertension. The donor was a 50-year-old female (167 cm, 75 kg, body mass index (BMI) 26.9 kg/m²), donation-after-circulatory-death type III, with a predicted total lung capacity ratio of 1. A simultaneous heart procurement was performed, resulting in the absence of an atrial cuff at the left superior pulmonary vein (PV). The transplant was uneventful despite limited exposure due to the patient’s tiny posture (168 cm, 42 kg, BMI 14.9 kg/m²) and bilateral breast implants.

**Figure 1:**
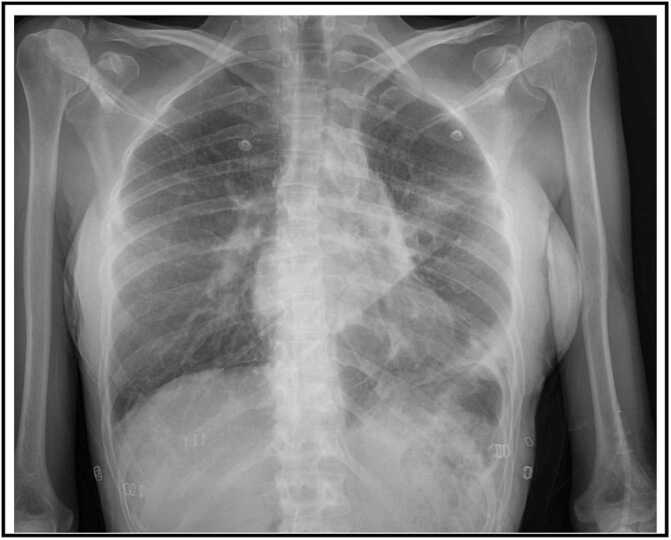
Chest X-ray 19 days post-transplant shows a vague shadowing in the left upper lobe.

**Figure 2:**
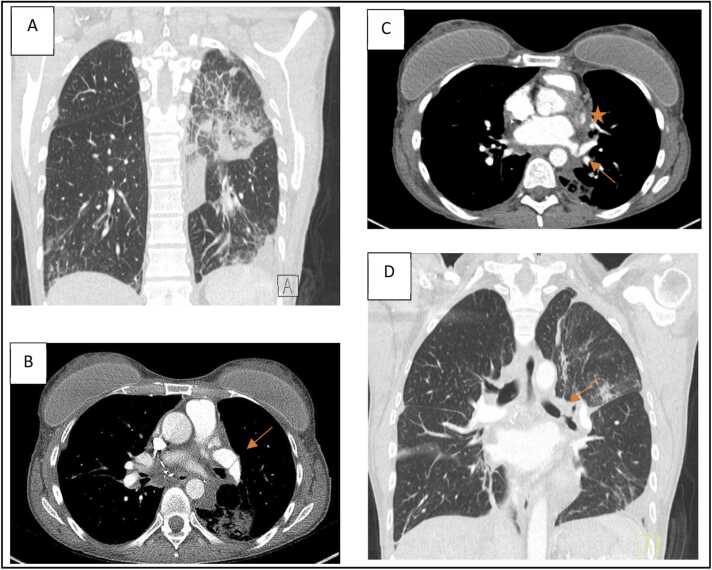
Chest computed tomography scan 20 days postoperatively. **A:** Infarction of left upper lobe; **B:** Kinking of the left main pulmonary artery (arrow); **C:** Occlusion of the left superior pulmonary vein (star) and kinking of the left inferior pulmonary vein (arrow); **D:** Stenosis at the left bronchial anastomosis (arrow).

**Figure 3:**
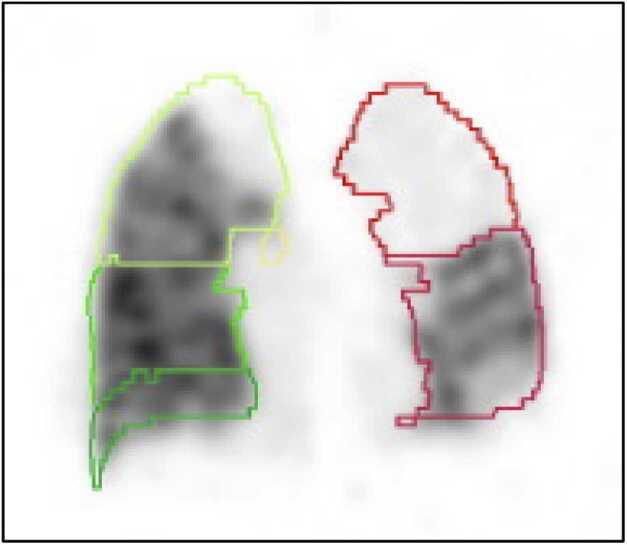
Chest ventilation/perfusion-scintigraphy 21 days postoperatively showing a left upper lobe mismatch with ventilation of the right lung 64% (14% upper, 12% middle, 38% lower lobe) versus left lung 36% (8% upper, 28% lower lobe), and perfusion of the right lung 73% (18% upper, 14% middle, 41% lower lobe) versus left lung 27% (2% upper, 25% lower lobe).

**Figure 4:**
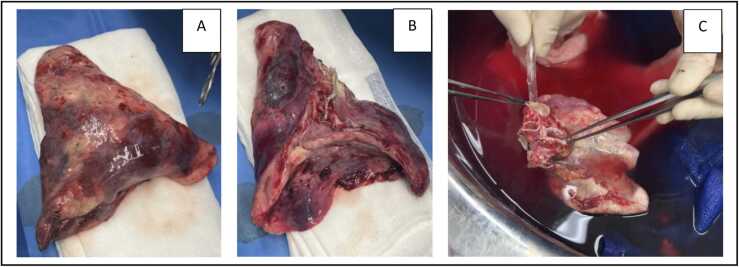
Macroscopic pictures after pneumonectomy and ex-situ upper lobe lobectomy. **A:** Anterior side of upper lobe post-lobectomy; **B:** Hilar side of upper lobe post-lobectomy; **C:** Antegrade flush with preservation solution of the lower lobe during the bench.

**Figure 5:**
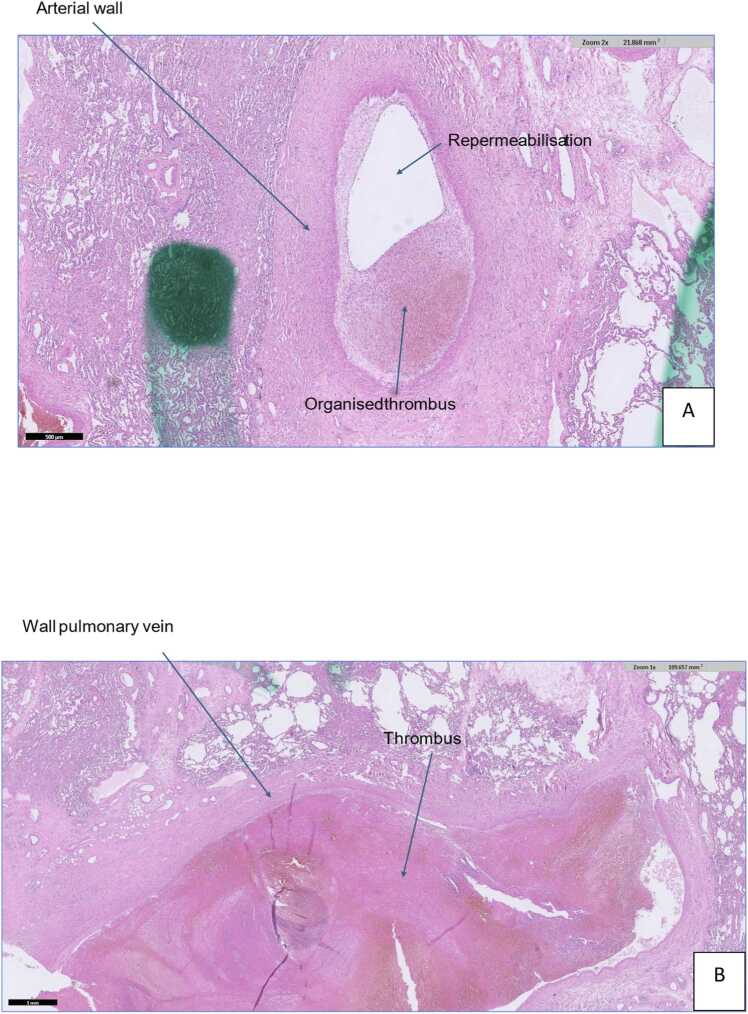
Histopathological findings consistent with infarction related to pulmonary artery thrombosis **(A)** and pulmonary vein thrombosis **(B)**.

**Figure 6:**
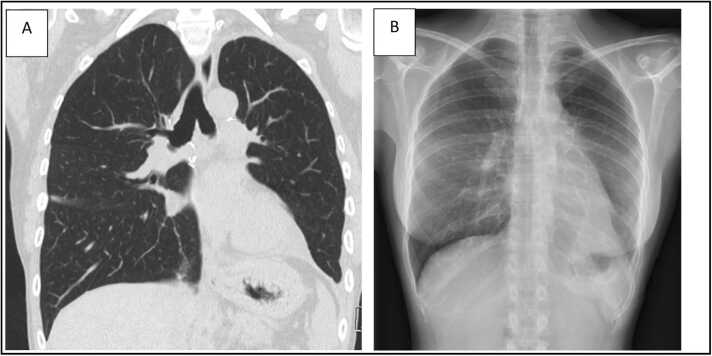
**A:** Chest computed tomography scan 162 days postoperatively; **B:** Chest X-ray 222 days postoperatively (superposition of bilateral breast implants).

Postoperative recovery was uncomplicated with extubation on postoperative day (POD) 1. However, due to an asymptomatic persistent left UL shadowing on chest X-ray at POD19 (Figure 1) in combination with a narrowed left bronchial anastomosis on bronchoscopy, additional imaging was done. Chest computed tomography (Figure 2) at POD20 showed on the left side UL infarction, kinking and narrowing of the main PA and inferior PV, occlusion of the superior PV, and stenosis at the bronchial anastomosis. Ventilation/perfusion scintigraphy (Figure 3) at POD21 revealed reduced ventilation (8%) and perfusion (2%) in the left UL. To prevent infection and clinical deterioration, a redo left exploratory thoracotomy with intrapericardial vascular control was performed. The anomalies at each anastomosis were confirmed: (i) PA stenosis caused by a constrictive tissue strand; (ii) superior PV thrombosis and inferior PV stenosis; and (iii) bronchial stenosis on the membranous part. A pneumonectomy at the suture lines was carried out, followed by cold ante- and retrograde flush. Multiple thrombi were rinsed from the superior PV and a consolidation of segment 2-4-5 was observed. Given the risk for abcedation, an ex-vivo upper lobectomy was performed (Figure 4) with closure of the UL bronchus. The lower lobe was reimplanted by anastomosing: (i) donor to recipient main bronchus; (ii) donor to recipient main PA; and (iii) donor inferior PV to recipient atrium. Implantation lasted 47 min and total ischemia 105 min. No transfusion or ECLS were required. Histopathology confirmed infarction due to PA and PV thrombosis, with no evidence of acute pneumonia (Figure 5). Postoperative course was uneventful with hospital discharge at POD20 after lobar-lung auto-reimplantation. At last follow-up, seven months postoperatively, the patient is in good clinical condition with a clear chest X-ray and CT-scan (Figure 6), and improving graft function (forced vital capacity 2.52 L or 64%pred, forced expiratory volume in one second 2.11 L or 67%pred).

## Discussion

Lobar-lung auto-reimplantation is a surgical technique avoiding pneumonectomy while addressing complex anastomotic issues, thereby maximizing the preservation of pulmonary function. This procedure has previously been described in the treatment of various lung diseases, including centrally located tumors, bronchial necrosis following radiotherapy, post-pneumectomy-like syndrome, and bronchopleural fistula after right upper bronchial sleeve lobectomy.^4^, ^5^, ^6^, ^7^, ^8^ The ex-vivo nature of the operation allows for thorough lung inspection, adequate flushing, targeted resection of the affected lung tissue, meticulous hilar preparation, and reduction of ischemia-reperfusion injury post-reimplantation.

To the best of our knowledge, this is the first case report describing lobar-lung auto-reimplantation following LTx, which we could attribute the term “auto-retransplantation.” Our prior experience with lobar LTx facilitated the execution of this technique. However, centers with limited expertise in autotransplantation or lobar LTx may need to consider transferring patients to specialized centers. Additionally, to minimize the risk of anastomotic complications post-LTx, a Clamshell thoracotomy should be considered when exposure is restricted.

## Conclusion

Lobar-lung auto-reimplantation may be considered an effective therapeutic option for managing complex anastomotic complications after LTx, leading to favorable short-term outcomes.

## Patient Consent

Authors confirm that appropriate patient consent to publish this case report was obtained (s69963).

## Declaration of Generative AI and AI-Assisted Technologies in the Writing Process

During the preparation of this work, the author(s) used ChatGPT (OpenAI) in order to review spelling and grammar. After using this tool/service, the author(s) reviewed and edited the content as needed and take(s) full responsibility for the content of the publication.

## Declaration of Competing Interest

The authors declare the following financial interests/personal relationships which may be considered as potential competing interests: DVR is Editor-in-Chief of JHLT Open and is supported by the Broere Charitable Foundation. RV is a senior Clinical Research Fellow of the Research Foundation-Flanders (FWO) (#1803521N). LJC is a senior Clinical Research Fellow of the Research Foundation-Flanders (FWO) (#18E2B24N) and is supported by a KU Leuven University Chair funded by Medtronic. The other authors declare that they have no known competing financial interests or personal relationships that could have appeared to influence the work reported in this paper.
